# Microbial Diversity and Enzyme Activity as Indicators of Permethrin-Exposed Soil Health

**DOI:** 10.3390/molecules28124756

**Published:** 2023-06-14

**Authors:** Agata Borowik, Jadwiga Wyszkowska, Magdalena Zaborowska, Jan Kucharski

**Affiliations:** Department of Soil Science and Microbiology, Faculty of Agriculture and Forestry, University of Warmia and Mazury in Olsztyn, 10-719 Olsztyn, Poland; agata.borowik@uwm.edu.pl (A.B.); m.zaborowska@uwm.edu.pl (M.Z.); jan.kucharski@uwm.edu.pl (J.K.)

**Keywords:** soil quality, metagenomics, insecticide, pyrethroids, microorganisms, enzymes, *Zea mays*

## Abstract

Owing to their wide range of applications in the control of ticks and insects in horticulture, forestry, agriculture and food production, pyrethroids pose a significant threat to the environment, including a risk to human health. Hence, it is extremely important to gain a sound understanding of the response of plants and changes in the soil microbiome induced by permethrin. The purpose of this study has been to show the diversity of microorganisms, activity of soil enzymes and growth of *Zea mays* following the application of permethrin. This article presents the results of the identification of microorganisms with the NGS sequencing method, and of isolated colonies of microorganisms on selective microbiological substrates. Furthermore, the activity of several soil enzymes, such as dehydrogenases (Deh), urease (Ure), catalase (Cat), acid phosphatase (Pac), alkaline phosphatase (Pal), *β*-glucosidase (Glu) and arylsulfatase (Aryl), as well as the growth of *Zea mays* and its greenness indicators (SPAD), after 60 days of growth following the application of permethrin, were presented. The research results indicate that permethrin does not have a negative effect on the growth of plants. The metagenomic studies showed that the application of permethrin increases the abundance of *Proteobacteria*, but decreases the counts of *Actinobacteria* and *Ascomycota*. The application of permethrin raised to the highest degree the abundance of bacteria of the genera *Cellulomonas*, *Kaistobacter*, *Pseudomonas*, *Rhodanobacter* and fungi of the genera *Penicillium*, *Humicola*, *Iodophanus*, *Meyerozyma*. It has been determined that permethrin stimulates the multiplication of organotrophic bacteria and actinomycetes, decreases the counts of fungi and depresses the activity of all soil enzymes in unseeded soil. *Zea mays* is able to mitigate the effect of permethrin and can therefore be used as an effective phytoremediation plant.

## 1. Introduction

Soil, one of the most important natural resources, is the landscape’s inherent component. It undergoes modifications over time, while storing and converting energy and matter [1]. According to the Natural Resources Conservation Service—USDA [2], soil health is defined as ‘the continued capacity of soil to function as a vital living ecosystem that sustains plants, animals, and humans’. Healthy soil ensures bountiful yields, clean waters and healthy forests. It plays a key role in production of safe food; it is also vital for sustainable and eco-friendly development and for nature conservation. Soil degradation and loss of soil quality may give rise to economic decline and social unrest [1,2].

Fertile soils are characterized by high microbiological activity [3,4]. One gram of soil can contain from hundreds of millions to billions of microorganisms [5,6]. Microorganisms interact with soil components, play a vital role in the biogeochemical cycle of elements and in the promotion of the growth and development of plants by supplying them with nutrients and phytohormones while inhibiting the development of pathogens [3,7]. The biomass of microorganisms depends on many factors, such as temperature, moisture, oxygen content, pH, methods of crop cultivation, genotypes of plants, the development of pathogens, the pressure of heavy metals and plant protection chemicals [8,9,10,11]. A crop cultivation technology incompatible with good agricultural practice, e.g., striving to maximize production at the lowest costs, can disrupt the soil homeostasis or relations between microorganisms and plant roots, thereby creating an environment that does not favor the development of plants [1]. 

According to simulations discussed in [12], the total sales of agricultural chemicals will increase in 2030 by ca. 22% relative to 2021, and will achieve the value of USD 279.12 billion. The global consumption of pesticides in agriculture in 2020 reached nearly 2.7 million metric tons, which corresponded to over 57% of the amount used in 1990; this total quantity included 606,000 tons of fungicides and bactericides as well as 471,000 tons of insecticides. Permethrin, classified as one of pyrethroids, is a synthetic-organic insecticides [7,13]. According to the Toxics Release Inventory (TRI) created by the Emergency Planning and Community Right-to-Know Act (EPCRA), supported by the U.S. Environmental Protection Agency (EPA), permethrin is a toxic substance hazardous to the environment. It has been registered by EPA since 1979, and is sold in many products, e.g., for fogging and pest control. The EPA report (2023) states that permethrin is used over an area of 39 million acres in the USA to control mosquitoes [1,14,15,16]. According to the data displayed on the website of the Environmental Protection Agency, the Food and Drug Administration, permethrin is most often used in the urban landscape [14,17,18,19]. 

Due to their lipophilic character, permethrin and its derivates can bioaccumulate in water [20], sediments [21,22], soils [23,24] and in organisms exposed to these substances [25]. According to the European Environment Agency (EEA) 2022 and FAO and UNEP (2021), our knowledge about the accumulation of persistent organic pollutants (POP), including insecticides, herbicides or fungicides in agricultural soils, is constantly being enriched. This progress is stimulated by the development of novel research methods, which, for example, enable us to study the consequences of DNA damage in invertebrates [26] or to trace changes in the soil microbiome [24,27]. 

In line with Directive 2009/128/EC, establishing a framework for Community action to achieve the sustainable use of pesticides, the European Commission’s strategy of ‘from field to table’ assumes that the use of agrichemicals should be reduced by 50% by year 2030. The need to examine the effect of permethrin on soil microorganisms, soil biochemical activity and growth of plants, and hence its impact on soil health, is further confirmed [28,29,30,31,32] since soil microorganisms, by participating in geochemical processes, play several functions in maintaining the soil’s structure. 

However, it should be emphasized that the biodegradation of pyrethroids is, to a large extent, related to their isomeric selectivity [33]. Pyrethroids possess 1–3 chiral centres and 2–8 stereoisomers, with the presence of chiral carbon atoms responsible for their enantioselective degradation [34]. Pyrethroids undergo biodegradation in the hydrolysis of the central ester bond, catalysed by carboxylesterase, the potential of which depends on the catalytic triad: glutamine, histidine and serine. The intermediates in the degradation of permethrin are, respectively: cyclopropane carboxylic acid, 3-phenoxybenzyl alcohol, 3-phenoxybenzaldehyde (PBAld), 1,2-benzenedicarboxylic acid or 1,2-benzenedicarboxylic butyl decyl ester [35,36]. The efficiency of permethrin degradation depends, to a large extent, on soil properties such as: moisture, soil texture, organic matter content, pH and temperature. Due to the lipophilic properties of pyrethroids, both organic matter and clay content control their bioavailability to microorganisms. In turn, the processes of adsorption and desorption of these compounds are significantly affected by pH and soil moisture [37,38].

The controlled insect species, after applying permethrin, may, as in the case of other insecticides, develop many different defence mechanisms that allow them to survive [7,39].

These mechanisms can be divided into physiological mechanisms involving changes in the rate of permeation and transport across membranes, biochemical ones consisting of changing or increasing detoxification metabolism, and behavioural ones consisting of avoiding the lethal dose of the insecticide used by the insect. All these resistance mechanisms are genetically determined and controlled by appropriate genes [7,39,40,41]. Permethrin, used several times during the season to control pests, mainly ticks, cockroaches or pharaoh ants, through continuous contact can lead to the permanent multiplication of insecticide-metabolizing bacteria, which is particularly important for the development and intensification of mosquito resistance to insecticides [39,40]. Maintaining proper relationships between the physical, chemical and biological properties of soil is fundamental to the proper quality of soil, which is crucial for life on our planet [1,5]. All of these three categories of soil characteristics are largely dependent on the content of organic matter in soil, which determines the soil’s biodiversity. Organic matter creates the base of the so-called soil food web [42,43], associated with the release of nutrients by microorganisms. There are different indicators that serve to evaluate the productivity and fertility of soil, but Doran and Zeiss [44] underline how difficult it is to develop such indices. According to the strategy of the LUCAS module of Soil Biodiversity and Pesticides [45], the determination of the biological diversity of soil can be achieved, for example, by sequencing specific DNA regions extracted and amplified for any type of an environmental sample. The aim of our study has been to present simple indicators for evaluation of the quality of soil exposed to the pressure of permethrin, a third generation insecticide. To achieve this aim, metagenomic and biochemical assays of soil were made. The effect of the application of permethrin on the growth and development of *Zea mays*, on the diversity of bacteria and fungi and on the activity of soil enzymes was examined.

## 2. Results

### 2.1. The Reaction of Bacteria and Fungi to Permethrin

#### 2.1.1. Non-Cultured Bacteria

**Figure 1:**
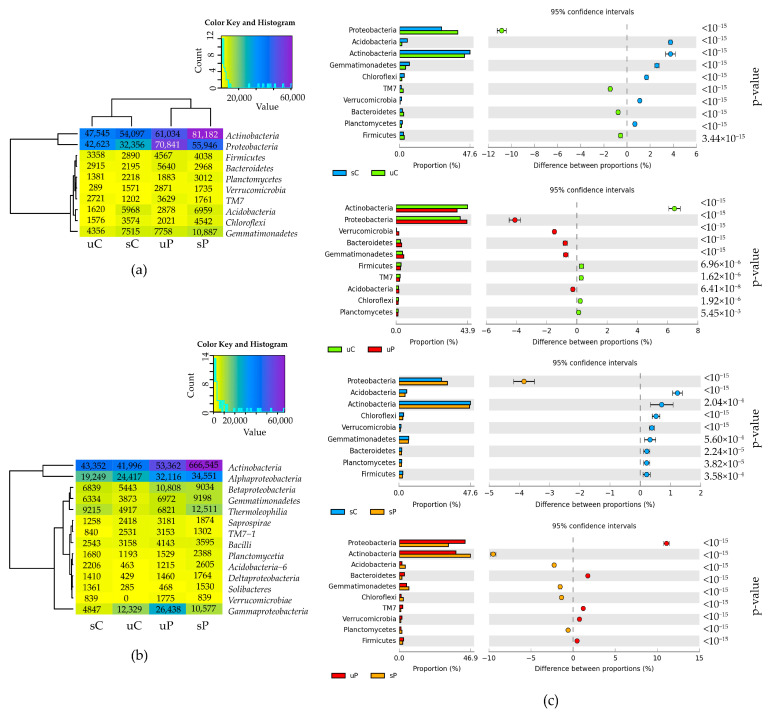
The relative abundance of dominant (**a**) bacterial types and (**b**) bacterial classes in soils, presented on a heat map. (**c**) The differences in the relative abundance proportions of bacterial types, presented using the STAMP statistical analysis software. sC—sown soil without permethrin, sP—sown soil with permethrin, uC—unsown soil without permethrin, uP—unsown soil with permethrin.

The monitoring of the soil’s biological diversity through the sequencing of 16S DNA amplicons showed that from 99.3% to 99.7% of sequences belonged to the kingdom Bacteria. In all soil samples, unsown and sown with *Zea mays*, the phyla *Actinobacteria* and *Proteobacteria* dominated among the 29 types. Another eight dominant types of bacteria, representing ≥ 1% of all acquired sequences, were the phyla *Gemmatimonadetes*, *Acidobacteria*, *Chloroflexi*, *Firmicutes*, *Planctomycetes*, *Bacteroidetes*, *Verrucomicrobia* and TM7 (Figure 1a,c). The cultivation of *Zea mays* (sC_uC) contributed to a decrease in the relative abundance of *Proteobacteria* by 10.8% and an increase in the relative abundance of *Actinobacteria* by 3.8% (Figure 1c). The pollution of unsown soil with permethrin (uC_uP) decreased the relative abundance of *Actinobacteria* by 6.5% and increased the relative abundance of *Proteobacteria* by 4.1%. Permethrin, when applied to the soil, which was cropped with *Zea mays* (sC_sP), decreased the relative abundance of *Proteobacteria* by 3.8% but did not considerably affect the abundance of *Actinobacteria*. The cultivation of *Zea mays* on soil polluted with permethrin increased the relative abundance of *Actinobacteria* by 9.5% and decreased *Proteobacteria* by 11.1% (uP_sP). The most frequently present classes of bacteria were *Actinobacteria* and *Thermoleophilia* of the phylum *Actinobacteria*; *Alphaproteobacteria*, *Gammaproteobacteria* and *Betaproteobacteria* of the phylum *Proteobacteria*; and *Gemmatimonadetes* of the phylum *Gemmatimonadetes* (Figure 1b). 

**Figure 2:**
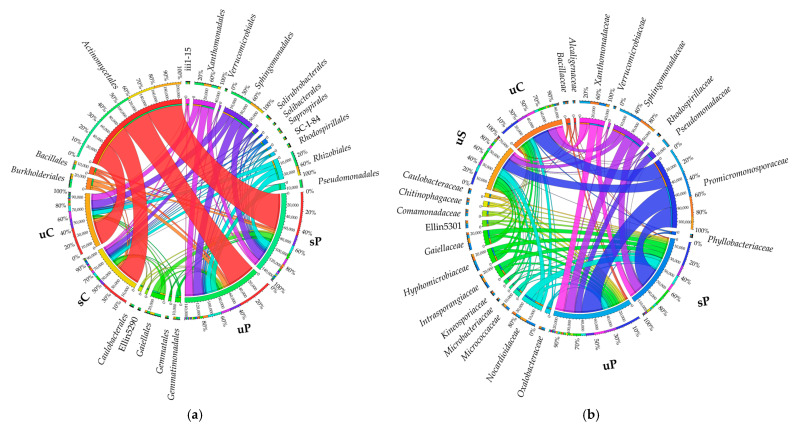
Orders (**a**) and families (**b**) of bacteria visualized with the help of a software package designed for data visualization in a circular layout (data refer to OUT ≥ 1%). sC—sown soil without permethrin, sP—sown soil with permethrin, uC—unsown soil without permethrin, uP—unsown soil with permethrin.

Once the sequences were assigned to subsequent taxonomic levels, it emerged that the orders *Actinomycetales*, *Sphingomonadales* and *Xanthomonadales* dominated in all analyzed soils (Figure 2a). Taking into account OUT ≥ 1%, the order *Actinomycetales* was represented by *Promicromonosporaceae, Nocardioidaceae*, *Intrasporangiaceae* and *Micrococcaceae*, the order *Sphingomonadales* was represented by *Sphingomonadaceae,* and the order *Xanthomonadales* was represented by *Xanthomonadaceae* (Figure 2b).

**Figure 3:**
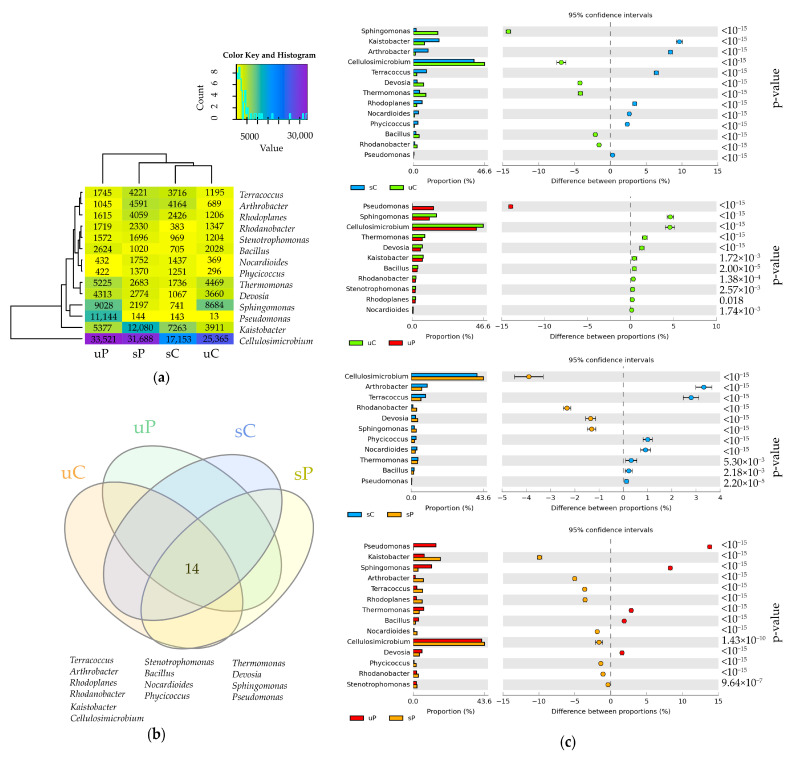
The relative abundance of dominant bacterial genera in soils (**a**) presented on a heat map, OUT ≥ 1%; (**b**) Venn diagram for bacterial genera, calculated from OUT ≥ 1% data; (**c**) differences in the relative abundance proportions of bacterial genera, presented using the STAMP statistical analysis software. sC—sown soil without permethrin, sP—sown soil with permethrin, uC—unsown soil without permethrin, uP—unsown soil with permethrin.

Regardless of the application of permethrin or sowing of *Zea mays*, the dominant bacteria in soil were the ones of the genera: *Cellulosimicrobium* classified to the family *Promicromonosporaceae*, order *Actinomycetales*, class *Actinobacteria*, phylum *Actinobacteria*; *Kaistobacter* classified to the family *Sphingomonadaceae*, order *Sphingomonadales*, class *Alphaproteobacteria*, phylum *Proteobacteria*; and *Sphingomonas* classified to the family *Sphingomonadaceae*, order *Sphingomonadales*, class *Alphaproteobacteria*, phylum *Proteobacteria* (Figure 3a).

After obtaining OTU data ≥ 1% at the genus level in all soil samples, the relative abundance data of bacterial genera indicated that the cultivation of *Zea mays* (sC_uC) contributed the most to a decrease in the abundance of bacteria of the genus *Cellulosimicrobium* (by 6.8%) and *Sphingomonas* (by 14.2%) and an increase in the abundance of *Kaistobacter* (by 9.6%) and *Arthrobacter* (by 8.4%). The application of permethrin (uC_uP) contributed the most to an increase in the abundance of bacteria of the genus *Pseudomonas* (by 13.9%) and a decrease in counts of *Cellulosimicrobium* and *Sphingomonas* (by 4.6%). The application of permethrin to soils sown with *Zea mays* (sC_sP) most significantly raised the relative abundance of *Cellulosimicrobium* (by 3.9%) and *Rhodanobacter* (by 2.3%), and decreased the relative abundance of *Arthrobacter* (by 3.3%) and *Terracoccus* (by 2.8%). The cultivation of *Zea mays* on soil polluted with permethrin (uP_sP) increased the relative abundance of bacteria *Kaistobacter* (by 9.9%), *Arthrobacter* (by 5%), *Terracoccus* and *Rhodoplanes* (by 3.6%), while decreasing the abundance of *Pseudomonas* (by 13.8%), *Sphingomonas* (by 8.3%) and *Thermomonas* (by 2.9%) (Figure 3c). 

The research results did not reveal any unique types of bacteria in the analyzed soils. In fact, all the identified species of bacteria comprised a shared microbiome of soils, polluted and unpolluted ones (Figure 3b). 

#### 2.1.2. Non-Cultured Fungi

**Figure 4:**
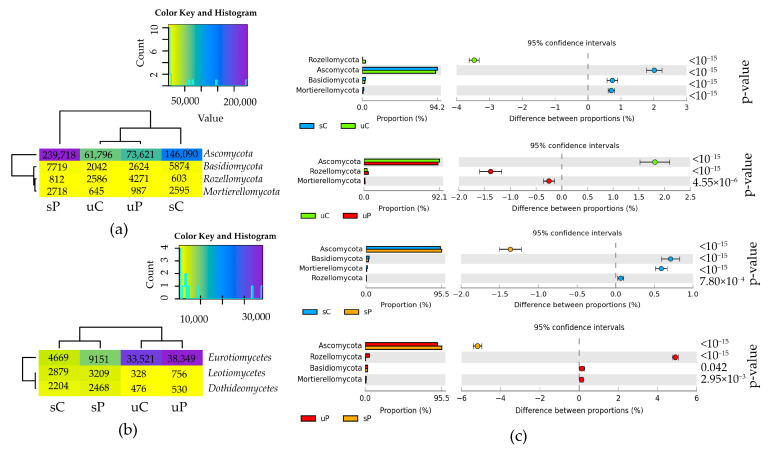
The relative abundance of dominant (**a**) bacterial types; (**b**) bacterial classes in soils, presented on a heat map; (**c**) differences in the relative abundance proportions of bacterial types, presented using the STAMP statistical analysis software. sC—sown soil without permethrin, sP—sown soil with permethrin, uC—unsown soil without permethrin, uP—unsown soil with permethrin.

The metagenomic analysis of fungi led to the identification of 60.7% to 70.8% of sequences with the OTU number ≥ 1% as belonging to the kingdom Fungi. In all soil samples, both unsown and sown with *Zea mays*, the phylum *Ascomycota* dominated among the 13 types. Other dominant types of fungi were *Basidiomycota*, *Mortierellomycota* and *Rozellomycota* (Figure 4a). The cultivation of *Zea mays* (sC_uC) contributed to an increase in the abundance of *Ascomycota* (by 2.0%) and a decrease in the abundance of *Rozellomycota* (by 3.5%). The pollution of soil under *Zea mays* with permethrin (sC_sP) increased the abundance of the phylum *Ascomycota* (by 1.4%). In soil polluted with permethrin, the cultivation of *Zea mays* (uP_sP) raised the abundance of *Ascomycota* (by 5.2%) and decreased that of *Rozellomycota* (by 4.9%) (Figure 4c). Most sequences of *Eurotiomycetes* classified to the class of fungi were determined in unsown soils and in sown soils polluted with permethrin. Seeding soils with *Zea mays* had an unambiguously more positive effect on *Leotiomycetes* and *Dothideomycetes* (Figure 4b).

**Figure 5:**
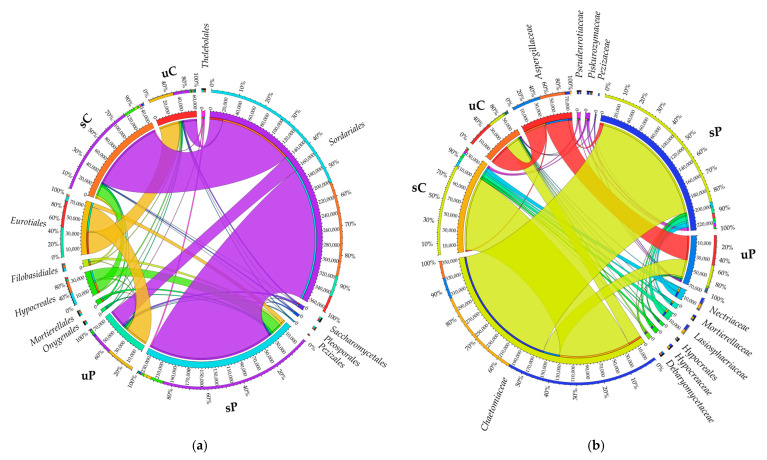
Orders (**a**) and families (**b**) of fungi visualized with the help of a software package designed for data visualization in a circular layout (data refer to OUT ≥ 1%). sC—sown soil without permethrin, sP—sown soil with permethrin, uC—unsown soil without permethrin, uP—unsown soil with permethrin.

Sequences of mold fungi classified to the order *Sordariales* were most abundant in sown soils, and those of the order *Eurotiales* in unsown soils (Figure 5a). Having assigned the sequences to the subsequent taxonomic levels, it was found that the dominant families of fungi were *Chaetomiaceae* of the order *Sordariales*, class *Sordariomycetes*, phylum *Ascomycota,* as well as *Aspergillaceae* which belong to the order *Eurotiales*, class *Eurotiomycetes*, type *Ascomycota*, with *Chaetomiaceae* (in 81–85%) dominating in soils under *Zea mays*, while *Aspergillaceae* (52–54%) dominated in unsown soils (Figure 5b). 

**Figure 6:**
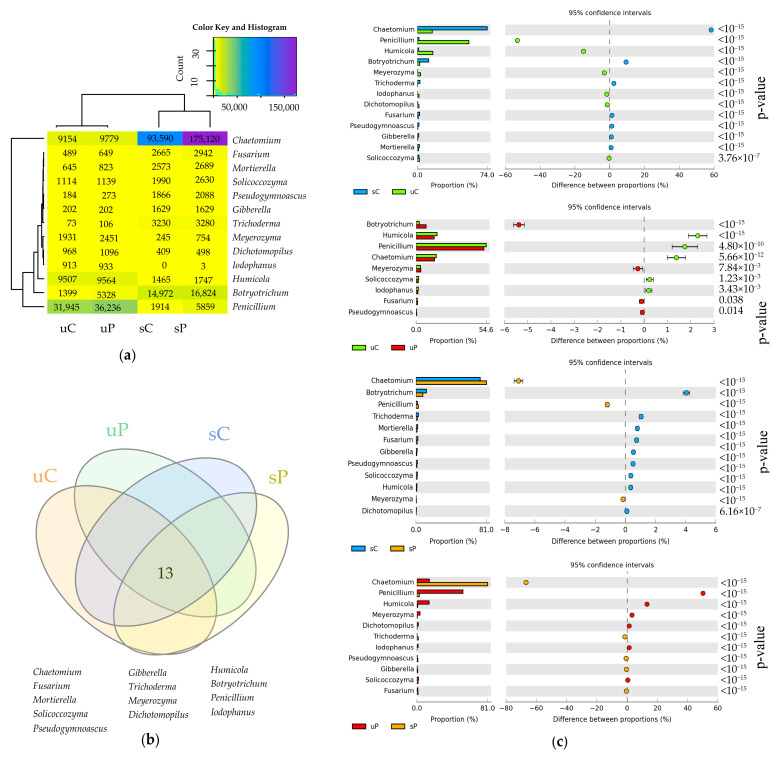
The relative abundance of dominant fungal genera in soils (**a**) presented on a heat map, OUT ≥ 1%; (**b**) Venn diagram for fungal genera, calculated from OUT ≥ 1% data; (**c**) differences in the relative abundance proportions of fungal genera, presented using the STAMP statistical analysis software. sC—sown soil without permethrin, sP—sown soil with permethrin, uC—unsown soil without permethrin, uP—unsown soil with permethrin.

In soils sown with *Zea mays*, the dominant fungi were the ones of the genus *Chaetomium* classified to the family *Chaetomiaceae* (Figure 6a). After obtaining OTU data ≥ 1% at the fungal genus level in all soil samples, the relative abundance of fungal genera data indicated that the cultivation of *Zea mays* and application of permethrin contributed the most to the changes in the abundance of *Botryotrichum*, *Chaetomium*, *Humicola*, *Penicillium* and *Trichoderma*. Sowing the soils with *Zea mays* (sC_uC) increased the abundance of *Chaetomium* by 58.3% and *Botryotrichum* by 9.4%, but decreased the abundance of *Penicillium* by 53.1% and *Humicola* by 15.1% (Figure 6c). The application of permethrin to soils not sown with *Zea mays* (uC_uP) increased the abundance of fungi of the genus *Botryotrichum* by 5.4% and decreased the abundance of *Humicola*, *Penicillium* and *Chaetomium* by 2.3%, 1.7% and 1.4%, respectively. The application of permethrin to soils sown with *Zea mays* (sC_sP) increased the abundance of *Chaetomium* by 7.1% and decreased the abundance of *Botryotrichum* by 4.0%. The cultivation of *Zea mays* after the application of permethrin (uP_sP) increased the abundance of *Chaetomium* by 66.8% while decreasing the relative abundance of *Penicillium* by 50.1% and *Humicola* by 13.1% (Figure 6c). Similarly in the case of types of bacteria, it was impossible to distinguish a type of fungi unique in a given soil because all fungi comprised the core microbiome (Figure 6b). 

### 2.2. Cultured Microorganisms

**Figure 7:**
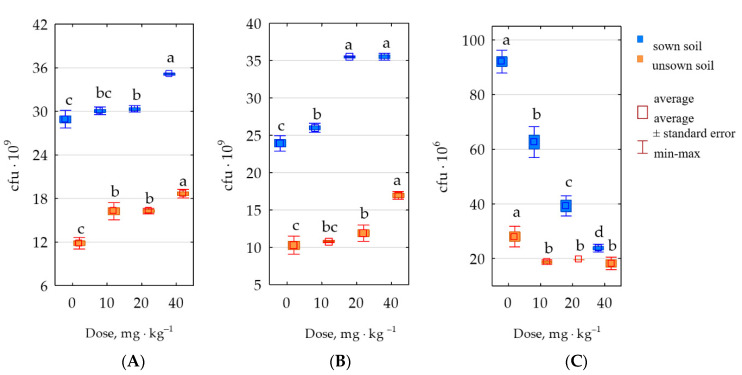
The abundance of (**A**) organotrophic bacteria, (**B**) actinomycetes, and (**C**) fungi in 1 kg of soil dry mass. Totals of 0–0 mg permethrin, 10–10 mg permethrin, 20–20 mg permethrin, 40–40 mg permethrin. Homogeneous groups (a–d) were created separately for sown soil and unsown soil.

The cultivation of *Zea mays* created suitable conditions for the development of organotrophic bacteria, actinomycetes, and fungi. Sowing the soils treated with permethrin raised the abundance of organotrophic bacteria by 51%, actinomycetes by 41% and fungi by 39%, on average, independent from the doses of permethrin. In the soil cropped with *Zea mays*, the presence of permethrin raised the counts of organotrophic bacteria in a range from 4% (10 mg permethrin) to 22% (40 mg permethrin); of actinomycetes from 9% (10 mg permethrin) to 48% (20 mg permethrin and 40 mg permethrin); and decreased the counts fungi from 32% (10 mg permethrin) to 74% (40 mg permethrin kg^−1^ d.m. of soil). In unsown soil, permethrin raised the counts of organotrophic bacteria from 37% (10 mg permethrin) to 58% (40 mg permethrin); actinomycetes from 5% (10 mg permethrin) to 65% (40 mg permethrin); and decreased the counts fungi from 30% (20 mg permethrin) to 35% (40 mg permethrin kg^−1^ d.m. of soil) (Figure 7). 

**Figure 8:**
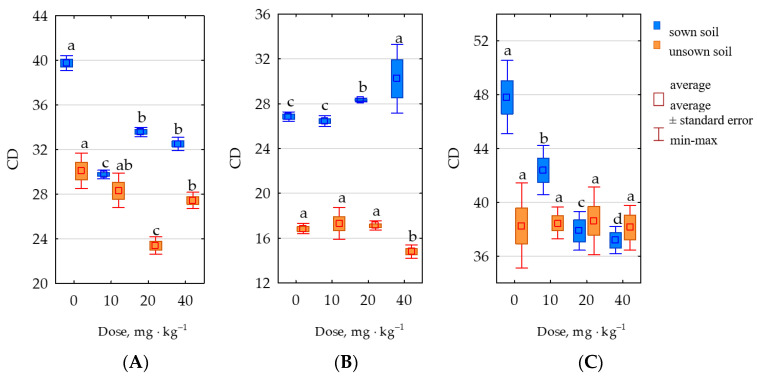
Colony development index (CD) of (**A**) organotrophic bacteria, (**B**) actinomycetes, and (**C**) fungi in 1 kg of soil dry mass. Totals of 0–0 mg permethrin, 10–10 mg permethrin, 20–20 mg permethrin, 40–40 mg permethrin. Homogeneous groups (a–d) were created separately for sown soil and unsown soil.

The cultivation of *Zea mays* increased the average colony development (CD) indices calculated for organotrophic bacteria (by 24%), actinomycetes (by 55%) and fungi (by 8%). Considering the applied doses of the insecticide, it can be concluded that the most significant negative impact on the CD index of organotrophic bacteria and actinomycetes in sown soil was produced by the lowest applied dose (10 mg permethrin), which depressed it by 25% and 15%, respectively, while the biggest decrease in the CD indices for fungi was induced by the highest dose of permethrin (40 mg permethrin), which lowered the CD index calculated from these microorganisms by 22%. In unsown soils, the CD index of organotrophic bacteria was most adversely affected by the medium dose of permethrin (20 mg permethrin), while the response of actinomycetes was most distinctly negative to the highest dose (40 mg permethrin) and no negative effect of the applied permethrin doses on mold fungi was observed (Figure 8).

**Figure 9:**
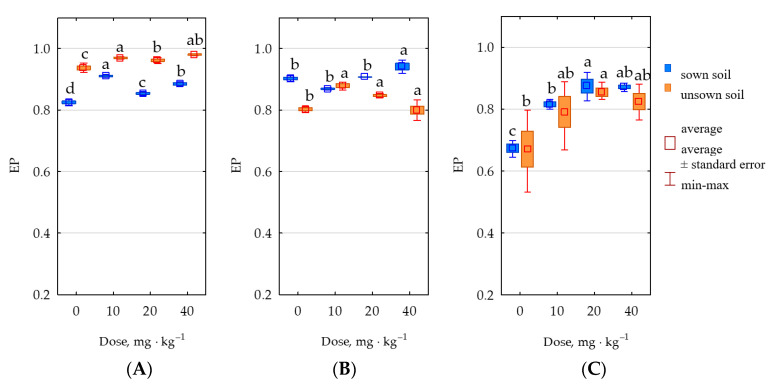
Ecophysiological diversity index (EP) of (**A**) organotrophic bacteria, (**B**) actinobacteria, and (**C**) fungi in 1 kg of soil dry mass. Totals of 0–0 mg permethrin, 10–10 mg permethrin, 20–20 mg permethrin, 40–40 mg permethrin. Homogeneous groups (a–d) were created separately for sown soil and unsown soil.

The ecophysiological diversity index (EP) showed that unsown soil was characterized by a higher diversity of organotrophic bacteria, while presenting lower diversity of actinomycetes and fungi (Figure 9). The mean EP indices for organotrophic bacteria were within the range of 0.869 in unsown soil to 0.963 in soil sown with *Zea mays,* for actinomycetes—from 0.888 in unsown soil to 0.909 in sown soil, and for fungi—from 0.786 in unsown soil to 0.809 in sown soil.

### 2.3. Response of Soil Enzymes to Permethrin

**Figure 10:**
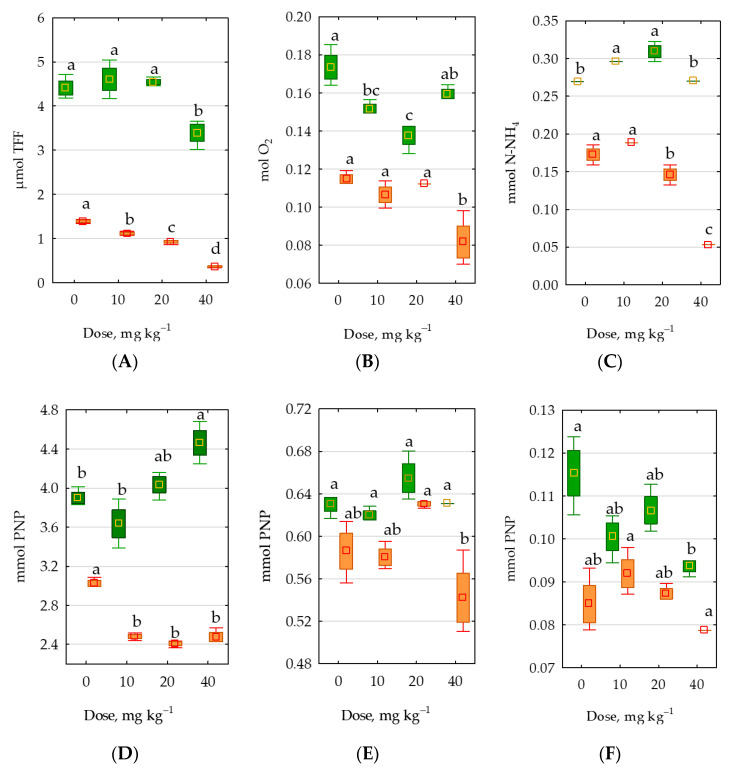

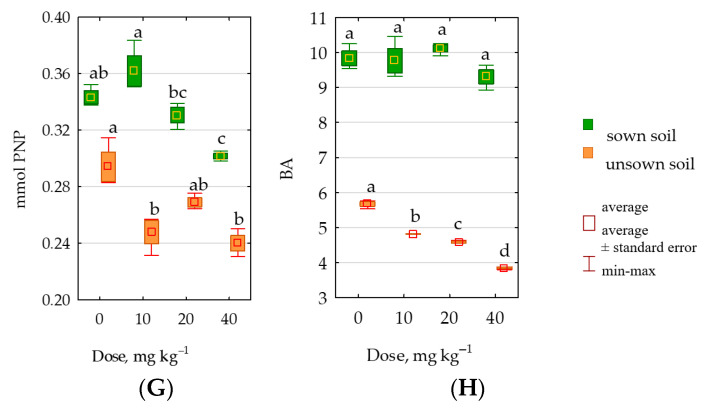
Enzymatic activity in 1 kg d.m. of soil h^−1^: (**A**) dehydrogenase, (**B**) catalase, (**C**) urease, (**D**) acidic phosphatase, (**E**) alkaline phosphatase, (**F**) arylsulfatase, (**G**) *β*-glucosidase and (**H**) biochemical activity coefficient (BA). Totals of 0–0 mg permethrin, 10–10 mg permethrin, 20–20 mg permethrin, 40–40 mg permethrin. Homogeneous groups (a–d) were created separately for sown soil and unsown soil.

The application of permethrin in the lowest dose (10 mg kg^−1^ d.m. of soil) was not shown to have a negative influence on most of the biochemical properties of the soil (Figure 10). Only the activity of acid phosphatase was significantly reduced in both unsown and sown *Zea mays* soil, as well as catalase in sown soil and *β*-glucosidase in unsown soil. The application of this preparation in an amount of 20 mg kg^−1^ d.m. of soil stimulated the activity of alkaline phosphatase and *β*-glucosidase in unsown soils as well as the activity of dehydrogenases, urease, alkaline phosphatase, acid phosphatase, in addition to which it raised the value of the biochemical soil quality index (BA) in soils under *Zea mays*. The highest tested permethrin dose (40 mg kg^−1^ d.m. of soil) exerted a negative effect in both sown and unsown soil on the activity of soil enzymes in both unsown soil and soil sown with *Zea mays*, with the exception of acid phosphatase in sown soil.

### 2.4. Response of Zea mays to Permethrin

**Figure 11:**
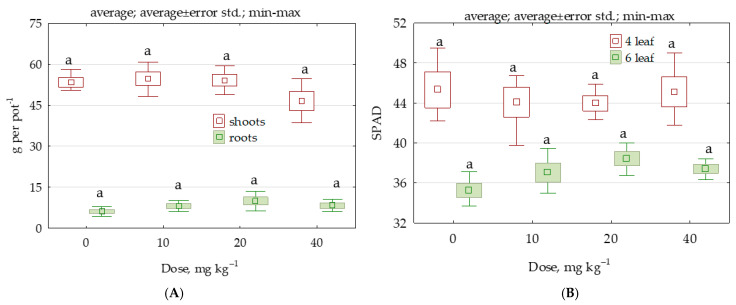
Results presenting (**A**) *Zea mays* yield in g dry weight per pot and (**B**) *Zea mays* SPAD.

Permethrin proved to be non-toxic to the test plant. Permethrin did not significantly decrease the yield of *Zea mays* nor did it lower the greenness indices that SPAD (ang. Soil and Plant Analysis Development) determined for *Zea mays* in the fourth and sixth leaf stage (Figure 11A,B). In brief, the growth and development of the test plant and the process of photosynthesis were undisturbed.

## 3. Discussion

### 3.1. Response of Non-Cultured Bacteria and Fungi to Permethrin 

Innovations in the protection of the quality of soils and crops should take advantage of the role of microbial communities, which can be a key element in the maintenance of soil health [46,47]. An evaluation of the quality of soil takes into account the biological diversity of organisms [48], and the biomass and activity of microorganisms and invertebrates [47,49,50]. In the course of this study, the 16S metagenomic analysis enabled us to identify from 109,188 to 176,303 OTUs of sequences of bacteria, and from 67,296 to 252,879 OTUs of fungi. The least OTUs of bacteria and fungi were identified in soils unsown and without permethrin, while the highest ones were determined in soils sown with *Zea mays* and treated with permethrin. The soils in this study were mainly colonized by bacteria of the types *Actinobacteria* and *Proteobacteria* and fungi of the phyla *Ascomycota* and *Basidiomycota*. These types of microorganisms, most active in soils polluted with pesticides, have also been identified in other studies [51,52].

According to Letourneau and Bothwell [53], a wide spectrum of pesticides contributes to the inhibition of harmful species. However, pesticides can also have an adverse impact on beneficial species. A selection induced by agrichemicals affects the competition among organisms in the soil environment, which consequently determines the values of the plant infestation indicators [54]. In our study, permethrin present in unsown soils and in soils sown with *Zea mays* stimulated the multiplication of all identified types of bacteria. The biggest changes in the proportions of the abundance of bacteria in unsown soils were detected in terms of the OTUs of bacteria of the type *Verrucomicrobia* and fungi *Rozellomycota*. In soils sown with *Zea mays*, bacteria of the type *Proteobacteria* and fungi of the type *Ascomycota* were the least resistant to permethrin. 

Most probably the most active types of bacteria in soils polluted with pesticides participating in their degradation are the bacteria of the genera *Pseudomonas* sp., *Stenotrophomonas* sp. [55], *Bacillus* sp. [56], *Serratia* sp. [57], *Acinetobacter* sp. [51,58], *Brevibacillus* sp. and *Sphingomonas* sp. [49], which partly lends credence to the obtained research results. The use of pesticide-degrading bacteria is the most promising strategy for the remediation of a soil environment contaminated with pyrethroids [33,58,59]. Regardless of the use of the soil and application of permethrin, our soils were colonized mainly by bacteria of the genus *Cellulosimicrobium* and fungi of the genus *Chaetomium*. Other microorganisms present in abundance were bacteria of the genera *Kaistobacter*, *Sphingomonas*, *Thermomonas* and fungi of the genus *Penicillium.* The bacteria which most probably decomposed permethrin in soil most effectively were the ones of the genera *Cellulosimicrobium* sp., *Kaistobacter* sp. and *Sphingomonas* sp. They appeared most numerously, which proves that they were most resistant to this pollutant. Our analysis of the soils not sown with *Zea mays* put the focus on the bacteria of the genus *Pseudomonas*, whose abundance increased by 100%. A significant increase in abundance was also noted for the bacteria of the genera *Arthrobacter*, *Terracoccus*, *Phycicoccus* and fungi *Botryotrichum*. In soils sown with *Zea mays*, bacteria of the genera *Rhodanobacter*, *Devosia*, *Rhodoplanes*, *Thermomonas*, *Stenotrophomonas* and fungi of the genera *Iodophanus*, *Meyerozyma* proved capable of removing the pollutant from soil. The metagenomic analysis allowed us to distinguish from 43,154 to 79,786 of sequences of bacteria ≥ 1% and from 58,524 to 216,065 of sequences of fungi. The smallest counts of assigned genera of bacteria were identified in soils sown with maize but not treated with permethrin (sC), while those of fungi—in soil not cropped with *Zea mays* without permethrin (uC). However, it should be emphasized that, the compilation of sowing the soil with *Zea mays* and 40 mg of permethrin kg^−1^ d.m. of soil contributed to reducing both the relative abundance of fungi and the development of their colonies, which was largely generated by the high dose of the applied insecticide. 

### 3.2. Response of Cultured Microorganisms

The improvement in the quality of soils consists mainly of raising the biomass of microorganisms [48,60]. In our study, the counts of cultured organotrophic bacteria and actinomycetes increased as doses of permethrin were higher. It can therefore be concluded that most microorganisms present in the soil could decompose permethrin quite effectively because pyrethroids can serve as a source of carbon for bacteria [48,60]. According to Imade and Babalola [61] and Bhatt [62], besides having a basic source of carbon, microorganisms also require other nutrients that facilitate the initial adaptation of bacteria to the environment, to accelerate their growth and to improve their capacity to degrade insecticides. Bokade et al. [63] concluded that strains of bacteria isolated from such an environment are helpful in the biomineralization of pollutants. The highest dose of the tested insecticide (40 mg permethrin) lowered the counts of fungi. Fungi are mainly acidophilic [64]. Thus, a decline in pH may have been caused by the desorption of residues of pesticides adsorbed on colloidal surfaces [65]. In the experiment reported in this article, the soil pollution with permethrin caused a moderate succession of microorganisms. It was only in the soil with the highest doses of the insecticides that a shift occurred between strategy k and strategy r microorganisms. Generally, the CD index reached higher values in soil cropped with *Zea mays.* Likewise, the EP index, which can assume values from 0 to 1, did not undergo drastic changes in response to the tested pyrethroid. Thus, it may be probable that the application of permethrin does not reduce the ecophysiological diversity of groups of microorganisms in soil. 

Microbial culture methods are commonly used, since the ability of microbial cultures to decompose organic compounds, sometimes toxic ones, to safer products does not adversely affect the quality of the soil environment [51,54,60,66]. Pesticide degradation by microorganisms usually proceeds in three stages: (I) the hydrolysis, oxidation or reduction of the primary compound; (II) the conjugation of the phase I metabolites with sugar or amino acids to increase their solubility in water and produce less toxic metabolites; and (III) the transformation of the phase II metabolites to secondary conjugates [67,68]. Most probably, the participation of microorganisms in the carbon and other nutrient cycles contributed, in our study, to the decomposition of permethrin, while the participation of microorganisms in processes of elevating the solubility of substances provided resources in the form of nutrients essential for the growth of plants, similar to *Zea mays* in our experiment.

### 3.3. Response of Soil Enzymes and Zea mays to Permethrin

The impact of insecticides on soil enzymes has not been thoroughly recognized yet [69]. Hence, complex studies that enable observations of changes in populations of microorganisms and enzymatic activity in the natural environment are particularly valuable [24,70]. Due to their structure, pyrethroids can be potentially hydrolyzed by carboxylesterase (EC 3.1.1.1) [71,72]. According to Bhatt et al. [73], it is esterases, also known as pyrethroid hydrolases and belonging to α/β proteins, that are responsible for the degradation of pyrethroids in the environment. The bioelimination of pyrethroids typically leads to breaking the ester bonds and the formation of carboxyl acids and alcohols [74,75]. Fang et al. [74] maintain that enzymes isolated from strains capable of degrading pyrethroids are close to lipases and esterases, which proves that microorganisms and their enzymes, with an effective capability of performing hydrolysis, play a key role in the elimination of residual amounts of pyrethroids. Our research results confirmed the growing counts of soil microorganisms responsible for the cycles of basic nutrients, i.e., C, N, P and S, in soil [76]. Pyrethroids are strongly bound to organic matter [58,77], which is of key importance for the maintenance of soil quality and productivity. 

The results of this study suggest that *Zea mays* can be used for the remediation of soils contaminated with pyrethroids. No negative effect of permethrin applied in doses from 10 to 40 mg on the growth of plants was detected. This could have been a consequence of plants being able to secrete pyrethroid-hydrolyzing enzymes [73]. However, it should be borne in mind that the application of excessively large quantities of pyrethroids can lead to a decrease in the uptake of water and nutrients, inhibit the photosynthesis of plants and disturb the hormonal balance [78]. According to Imade et al. [61], the positive effect of the grown plant *Zea mays* can be attributed to the plant’s increased secretion of organic compounds into soil. 

## 4. Materials and Methods

### 4.1. Soil Characterization

This study was conducted on soil which, according to the International Union of Soil Sciences and the United States Department of Agriculture soil classification, represented loamy sand. The soil was sampled from the Olsztyn Lake District (NE Poland, 53.72° N, 20.42° E). In the natural state, this was proper brown soil. A more specific description of the soil is presented in Table S1. A detailed description of the methods and laboratory equipment used for completing physicochemical and chemical assays of soil can be found in our previous paper [79].

### 4.2. Permethrin Characterization

Permethrin [3-(2,2-dichorovinyl)-2,2-dimethylcyclopropanecarboxylate] (number CAS: 52645-53-1), C_21_H_20_Cl_20_O, molecular weight −391.3 g mol^−1^ is a synthetic-organic chemical compound which belongs to pyrethroids [80]. In this experiment, it was applied in the form of the preparation Aspermet 200 EC (Asplant-Skotniccy Sp. J, Jaworzno, Poland), which contains 200 g of active substance, permethrin (P), per 1 dm^3^. As recommended, the preparation should be applied as 1% aqueous solution, in a dose of 10 dm^3^ of the solution per 200 m^2^ of area. When used outdoors, the preparation should be prepared as a 5% solution. 

### 4.3. Design of the Experiment

The experiment was conducted in a greenhouse at the University of Warmia and Mazury in Olsztyn (Poland). The experimental variants were prepared in polyethylene pots with the capacity of 3.5 dm^3^. The following doses of permethrin were tested: 0 mg, 10 mg, 20 mg and 40 mg per 1 kg d.m. of soil. Having thoroughly mixed permethrin with soil, and after placing batches of soil in the pots, the soil moisture content was increased to 60% of water capacity. The control consisted of unpolluted soil. In order to gain better understanding of the effect of permethrin on the soil microbiome, the experiment was conducted in two series: (1) unsown soil and (2) soil sown with *Zea mays* var. LG 32.52 (a variety registered in the European Union). After germination, the maize plants were thinned to 4 plants per pot. Throughout the experiment, water was replenished 2–3 times a day to maintain the set constant moisture content. Each variant was set up with four replications. The experiment lasted 60 days (June–August 2020). The length of daylight at that time of year ranged from 15 h 13 min to 16 h 35 min. The average air temperature was 17.9 °C in June to 19.8 °C in August. The average relative sir humidity was 77% (https://obserwator.imgw.pl) (accessed on 8 September 2022). 

In the fourth leaf (B) and sixth leaf development stage (BBCH 19), according to the SPAD leaf greenness index (Soil and Plant Analysis Development), was determined with a Chlorophyll Meter 2900P SPAD 502 (KONICA MINOLTA, Inc., Chiyoda, Japan). In BBCH 51 stage (beginning of tassel emergence), the yield of aerial parts and roots of maize was determined, the plants were cut, fragmented and dried in a dryer type Binder D-78532 Tuttlingen, Germany at a temperature of 60 °C for four days.

### 4.4. Methods of Soil Microbiological Analysis

#### 4.4.1. Breeding Microorganisms

Isolation of microorganisms was conducted through a series of dilutions, according to the method described in our previous paper [79]. Counts of microorganisms were determined as follows: organotrophic bacteria on Bunt and Roviry medium (1955) [81], actinomycetes on Kuster and Williams medium (1971), with addition of nystatin and antidyon (according to Parkinson 1971), and fungi on Martin medium (1950). All determinations were run in six replicates for each experimental object, all in moist soil. Microbial cultures were incubated in an incubator by Selecta Incudigit (Barcelona, Spain) at 28 °C for 10 days. Colony-forming units (c.f.u.) of microorganisms were presented per 1 kg^−1^ d.m. of soil. 

#### 4.4.2. Isolation of DNA and Identification of Bacteria and Fungi Using NGS Method

Genomic Mini AX Bacteria+” (A&A Biotechnology, Gdynia, Poland) served for isolation of DNA from soil samples, while employing universal starters 1055F (5′-ACGGGCGGTGTGTAC-3′) and amplifying a fragment of the bacterial genes 16S rRNA and ITS. Detailed PCR settings were presented in our earlier papers [82]. Sequencing of genetic material on the basis of the hypervariable region V3–V4 of the gene rRNA and the ITS1 fragment was carried out on a sequencer Illumina MiSeq (Genomed S.A. Warsaw, Poland). Primers 341F (5′-CCTACGGGNGGCWGCAG-3′), 785R (5′-GACTACHVGGGTATCTAATCC-3′) (Bacteria) and ITS1FI2 (5′-GAACCWGCGGARGGATCA-3′), 5.8S (5′-CGCTGCGTTCTTCATCG-3′) (Fungi) were used for amplification of the selected region. Sequences of bacteria and fungi were deposited in the GenBank NCBI under the access numbers: https://www.ncbi.nlm.nih.gov/nuccore/?term=OP914644:OP916021[accn] (accessed on 4 December 2022), https://www.ncbi.nlm.nih.gov/nuccore/?term=OP897054:OP897145[accn] (accessed on 2 December 2022), https://www.ncbi.nlm.nih.gov/nuccore/?term=OP978693:OP979103[accn] (accessed on 14 December 2022).

### 4.5. Biochemical Analysis of Soil

Determinations of the activity of dehydrogenases (Deh), catalase (Cat), urease (Ure), alkaline phosphatase (Pal), acid phosphatase (Pac), arylsulfatase (Aryl) and *β*-glucosidase (Glu) were performed with the methods presented in the papers [43,83]. The assays were carried out according to the methods by Öhlinger (1996), Johnson and Temple (1964) and by Alef and Nannipieri (1998). The assays for each research object were conducted in 3 replications, immediately after the soil samples were delivered to the laboratory. The activity of the analyzed enzymes was expressed in the following units: dehydrogenases µmol TFF kg^−1^ d.m. gleby h^−1^, catalase—mol O_2_ kg^−1^ d.m. gleby h^−1^, urease—mmol N-NH_4_ kg^−1^ d.m. gleby h^−1^, alkaline phosphatase, acid phosphatase, arylsulfatase and *β*-glucosidase—mmol PNP kg^−1^ d.m. gleby h^−1^. The activity of enzymes, except that of catalase, was determined on a spectrophotometer Perkin-Elmer Lambda 25 (Peabody, MA, USA).

### 4.6. Data Analysis and Statistical Processing

On the basis of the counts of the above groups of microorganisms, the colony development (CD) index [84] and the ecophysiological diversity (EP) [85] index for the microorganisms were calculated. Following the guidelines of the formula proposed by Sarathchandra et al. [84], each day, the colony growth of the incubated groups of microorganisms was consistently counted over a period of 10 days. The data were processed statistically in Statistica 13.1 [86]. Normality of distribution was verified with the Kruskal–Wallis test, and the results were submitted to the Duncan’s post hoc test. All data were displayed graphically, having eliminated OTUs lower than 1% in relation to the total number of OTUs. The types and genera of bacteria and fungi were statistically compared using the G-test (w/Yates’) + Fisher test, with the aid of the software STAMP 2.1.3 [87], and shown as heat maps in the software RStudio v1.2.5033 [88] with the gplots library [89] and R core [90]. Classes and orders of bacteria and fungi were analyzed in a circular layout in a software package Circos 0.68 [91]. For the visualization of unique data and shared genera of bacteria and fungi, the InteractiVenn software for analysis of sets was used [92].

## 5. Conclusions

Permethrin, applied in doses from 10 to 40 mg kg^−1^ d.m. of soil, did not demonstrate any negative effect on the growth of *Zea mays* or on the plant’s greenness index. The metagenomic assays showed that the application of permethrin increases the abundance of *Proteobacteria*, but decreases that of *Actinobacteria* and *Ascomycota.* The application of permethrin increased, to the highest degree, the abundance of bacteria of the genera *Cellulomonas*, *Kaistobacter*, *Pseudomonas* and *Rhodanobacter* and fungi of the genera *Penicillium*, *Humicola*, *Iodophanus* and *Meyerozyma*. It has been discovered that permethrin stimulates the multiplication of organotrophic bacteria and actinomycetes, depresses the CD index and elevates the EP index of organotrophic bacteria and fungi, while increasing the CD and decreasing the EP of actinomycetes. Permethrin lowers the activity of all analyzed enzymes, and the soil’s biochemical activity index, in unsown soils. Microorganisms present in the topsoil, from 0 to 20 cm depth, following the application of permethrin, adapt to changes occurring in the soil environment. Sowing the soil with *Zea mays* alleviates the stress induced by the application of permethrin, which eventually leads to the restoration of the soil quality. The influence of pyrethroids on the quality of soil can be estimated by analyzing changes in the assemblages of the soil microflora.

## Data Availability

The data presented in this study are available on request from the corresponding author.

## Supplementary Materials

The following supporting information can be downloaded at: https://www.mdpi.com/article/10.3390/molecules28124756/s1, Table S1. Properties of soil.

## Abbreviations

The following abbreviations are used in this manuscript: sC—sown soil without permethrin; sP—sown soil with permethrin; uC—unsown soil without permethrin; uP—unsown soil with permethrin, SPAD—greenness index; Deh—dehydrogenases; Cat—catalase; Ure—urease; Pac—acid phosphatase; Pal—alkaline phosphatase; Aryl—arylsulfatase; Glu—*β*-glucosidase.
