# Supplementary material for: Microbial Diversity and Enzyme Activity as Indicators of Permethrin-Exposed Soil Health
Source: Molecules. 2023 Jun 14;28(12):4756. doi: 10.3390/molecules28124756 (PMC10301950; doi:10.3390/molecules28124756)
Supplement: Supplementary file 1 [file molecules-28-04756-s001.zip › molecules-2391512-supplementary.pdf]

molecules

*Supplementary materials*

# Microbial Diversity and Enzyme Activity as Indicators of Permethrin-Exposed Soil Health

**Agata Borowik, Jadwiga Wyszkowska *, Magdalena Zaborowska and Jan Kucharski**

Department of Soil Science and Microbiology, Faculty of Agriculture and Forestry, University of Warmia and Mazury in Olsztyn, 10-719 Olsztyn, Poland; agata.borowik@uwm.edu.pl (A.B.); m.zaborowska@uwm.edu.pl (M.Z.); jan.kucharski@uwm.edu.pl (J.K.)

* Correspondence: jadwiga.wyszkowska@uwm.edu.pl

**Table S1.** Properties of soil.

| **Parameter** | **Content / Unit *** |
|---|---|
| Sandy loam | clay - 1.69 % fraction <0.002 mm<br>silt - 20.13% fraction 0.002-0.05 mm<br>sand - 78.18% fraction 0.05 mm-2.0 mm |
| $pH_{KCl}$ | 6.3 |
| hydrolytic acidity (HAC) | 1.16 cmol(+)g $kg^{-1}$ |
| sum of exchangeable base cations (EBC) | 5.82 cmol(+)g $kg^{-1}$ |
| cation exchange capacity (CEC) | 6.98 cmol(+)g $kg^{-1}$ |
| alkaline cation saturation (ACS) | 83.4% |
| $C_{org}$ | 7.5 g $kg^{-1}$ |
| $N_{tot}$ | 0.64 g $kg^{-1}$ |
| $P_{available}$ | 98 mg $kg^{-1}$ |
| $K_{available}$ | 133 mg $kg^{-1}$ |
| $Mg_{available}$ | 42 mg $kg^{-1}$ |

* all soil properties are given in dry weight (d.m.) of soil.
